# Effects of replacing a concentrate-based regimen with low-value date fruit on growth performance, meat quality, and fatty acid composition in Hamra lambs

**DOI:** 10.5194/aab-68-641-2025

**Published:** 2025-10-30

**Authors:** Nabila Berrighi, Özlem Aslan

**Affiliations:** 1 Laboratory of Biotechnology Applied to Agriculture and Environmental Preservation, Higher School of Agronomy, 27000 Mostaganem, Algeria; 2 TUBITAK MRC Life Sciences, Barış Mah. No: 1 P. K. 21, 41470 Gebze Kocaeli, Türkiye

## Abstract

This research investigates the effects of two different dietary regimes on body weight, fatty acid composition, vitamin E levels, and meat color attributes. The research involved two groups of Hamra breed lambs raised on pasture at the same farm in the El Bayadh area. The first group was supplemented with date fruit from two varieties, whole pitted date and Degla-Beida, while the second group was fed a concentrate diet. At the end of experiments, the lambs' weight gain was recorded, and *Longissimus thoracis* muscles were excised from each carcass. The results showed significantly higher weight gain ( p<0.05 ) in lambs fed a concentrate diet (Group 2) compared to the group fed date fruit (Group 1) (34.83 kg vs. 29.15 kg). However, the latter group displayed significant improvements in meat quality parameters, including a healthier fatty acid profile, with higher levels of polyunsaturated fatty acids and omega-3 fatty acids (16.38 %), particularly alpha-linolenic acid (0.30 %). Meat from lambs fed date fruit also showed elevated vitamin E content (1.47 mg/100 g) and stronger antioxidant capacity (1.14 mg TE/100 g), reducing lipid oxidation (0.05 mg MDA per kg meat) and enhancing meat stability. Group 1 also exhibited darker meat coloration and lower lipid oxidation rates. It is critical to highlight the unique advantages of the El Bayadh ecosystem for outdoor sheep breeding, which produces healthier meat that is rich in vitamin E and possesses an appealing color that could potentially enhance its marketability.

## 1 Introduction

The rising interest in the benefits of outdoor rearing and natural feeding systems for livestock has led to extensive investigations into the influence of natural diets on meat quality, particularly regarding the fatty acid composition. Previous research by Zong et al. (2016) has shown that beneficial fatty acids contained in meat significantly increase with the substantial consumption of herbs. In fact, herbivorous diets result in meat with an omega-3 fatty acid content that is 2.4 times greater than that found in meat produced using standard feeds. These beneficial fatty acids not only contribute to a healthier product for human consumption but also impart a more vibrant color to the meat. Indeed, the overall acceptance of meat products is greatly influenced by their color, a key factor for both appearance and marketability. Lipid oxidation rate in meat, impacting its color and shelf stability, is determined by intrinsic properties of lipids and the proportion of antioxidants, which are contingents on the diet of animals (Bekhit et al., 2019). Prache et al. (2011) advocate that pasture grazing stands as an optimal strategy for elevating antioxidant concentrations in sheep and lamb meat, thereby improving its nutritional quality. Utilizing by-products or co-products from agricultural sectors that are rich in health-boosting fatty acids and antioxidants might offer a sustainable approach to address nutritional challenges (Li et al., 2024). Among the natural antioxidants, vitamin E stands out, with α -tocopherol being the most biologically active isomer known for its effective antioxidant properties. In response to a growing consumer preference for natural food, it is important to implement measures that enhance livestock products' quality. Date fruit is considered a potential source of oleic acid (C18:1), the predominant fatty acid it contains. Oleic acid is recognized for its nutritional value, particularly its beneficial effects in reducing cardiovascular diseases by lowering total cholesterol and low-density lipoprotein cholesterol (Riley et al., 2022). As a result, they have been explored in numerous studies to develop potential functional food products or as a substitute for commercial oils. In Algeria, approximately 13% of the land comprises Sahara rangelands, which serve as primary grazing areas in livestock production (Senoussi et al., 2020). These high Sahara plains are characterized by a diverse ecosystem of drought-resistant herbaceous and shrubby plant species, such as *Stipa grostis* and *Panicum turgidum*, along with numerous forage shrubs like acacias, capable of thriving in arid and nutrient-poor soils (Khenfer et al., 2020). Over the past decade, Algeria has witnessed the emergence of innovative approaches to managing agricultural by-products within the framework of a circular economy. Among these initiatives, the utilization of date production waste as a sustainable feed source for livestock has gained attention (EL-Mously et al., 2023). This practice not only addresses the issue of agricultural waste management but also contributes to reducing the reliance on conventional feed resources, thus aligning with global efforts to promote sustainable agriculture and mitigating the environmental impact of food production systems. The amount of date palm waste produced annually is estimated to be around 200 000 t (FAO, 2016), and using it in animal feed promises both financial and health benefits (Rekis et al., 2020). Red meat constitution is affected by several elements, such as nutrition and farming methods. Utilizing by-products or co-products from agricultural sectors that possess a mix of health-boosting fatty acids (HBFAs) and antioxidants offers a sustainable approach to addressing nutritional issues. Among the natural antioxidants, vitamin E stands out, with α -tocopherol being the most biologically active isomer known for its potent antioxidant properties. The overall acceptance of meat products is greatly influenced by their color, a key factor for both appearance and marketability. This attribute is influenced by several factors including the species and breed of the animal, the dietary regime, body weight (BW) at the time of slaughter, the type of muscle, and the duration of meat aging (Prache et al., 2011). In response to a growing consumer preference for natural food, it is important to implement measures that enhance livestock products quality. The effects of feeding livestock with date palm by-products on meat quality have been documented (Rabee et al., 2022). However, further research is required to evaluate the effects of such diets on nutritional composition and sensory characteristics of meat for human consumption. With the aim to implement strategies to enhance the quality of livestock-derived products, an experimental investigation was conducted to evaluate the effects of replacing conventional concentrated feed with date fruit, particularly in regions like the Sahara where grazing resources are limited. The research focused on assessing the impact on lamb body weight gain, fatty acid profile, and vitamin E levels in the meat.

## 2 Materials and methods

### 2.1 Ethics approval

This experiment had no adverse effects on animal welfare. All procedures were approved by the Animal Science Committee of the National Institute of Agronomy of Algeria.

### 2.2 Experimental design: animal and feeding diet

**Figure 1:**
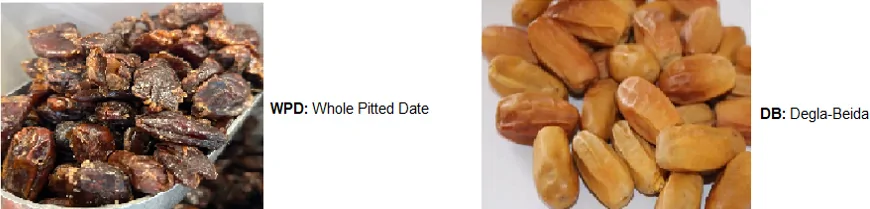
Date fruit varieties.

In this study, 50 males (10 months old) of Hamra (Deghma) lambs that were purchased in the southern oasis were earmarked for the study. These animals were evenly split into two groups of 25 units, each with an average BW of 32.2±2.4 kg. Lambs in Group 1 were fed date fruit (DF) of the Degla-Beida (DB) and whole pitted date (WPD) varieties (Fig. 1), while those in Group 2 received concentrate feed to achieve the desired commercial body weight. Throughout the study, each animal was housed individually in pens equipped with feeding and watering facilities. All animals were provided with unrestricted access to water. Details on diets are presented in Table 1.

**Table 1:** Ingredients' chemical composition and fatty acids (%, as feed basis) of the experimental diets used for sheep breeding at El Bayadh farm.

	DF		C	SEM	p value	
	DB	WPD				
Moisture (%)	10.97^d^	14.99^c^	07.26^a^	3.62	0.001^**^	
Crude ash (%)	02.60^b^	02.08^c^	03.11^a^	1.41	0.001^**^	
Crude protein (%)	04.33^d^	05.06^c^	12.45^b^	3.17	0.003^**^	
Crude fat (%)	0.28^c^	0.62^b^	05.61^a^	0.52	0.002^**^	
Crude fiber (%)	03.20^c^	04.49^b^	01.16^c^	0.32	0.000^***^	
Polyphenols (mg GAE/g DM)	660.65^a^	529.51^c^	110.27^d^	6.47	0.000^***^	
Vitamin E (mg/100 g)	01.83^b^	01.74^b^	01.02^c^	0.11	0.000^***^	
Fatty acids (%)						
C6:0	0.06	0.14	0.25	0.19	0.152^NS^	
C8:0	0.09	0.24	0.10	0.16	0.144^NS^	
C10:0	0.24	0.29	–	0.32	0.138^NS^	
C12:0	0.41	0.99	0.01	0.64	0.108^NS^	
C14:0	01.40	10.06	01.22	1.44	0.204^NS^	
C15:0	0.32	0.21	0.10	0.41	0.101^NS^	
C16:0	17.94^c^	33.57^a^	26.03^a,b^	3.74	0.023^**^	
C16:1	0.46^a,b^	0.69^a^	0.23^b^	0.15	0.003^**^	
C17:0	0.34^a^	0.20^b^	0.12^c^	0.06	0.002^**^	
C18:0	05.85^b^	08.51^a^	02.54^c^	2.43	0.000^***^	
C18:1 n-9t	0.15^b^	0.06^c^	–	0.11	0.000^***^	
C18:1 n-9c	63.28^a^	28.98^b^	34.13^c^	4.55	0.000^***^	
C18:2c n-6	06.78^c^	19.59^b^	29.39^a^	1.75	0.000^***^	
C18:3 n-3	0.30^b^	0.30^b^	01.28^c^	0.06	0.001^**^	
C20:0	0.31	0.54	0.41	0.21	0.187^NS^	
C20:1 n-9	0.12	0.07	0.79	0.23	0.166^NS^	
C21:0	0.38^b,c^	0.84^b^	0.07^c^	0.16	0.000^***^	
C22:0	0.06^b^	0.05^b^	0.23^a^	0.14	0.001^**^	
C22:1 n-9	–	0.15^a^	0.07^b^	0.07	0.001^**^	
C22:5 n-3	–	–	0.42	0.09	0.28^NS^	
C22:6 n-3	–	–	0.34	0.04	0.11^NS^	
C23:0	0.09^c^	2.24^b^	0.06^d^	0.13	0.000^***^	
C24:0	0.11^b^	–	0.22^a^	0.12	0.000^***^	
n-6	06.78^c^	19.59^b^	29.39^a^	1.75	0.000^***^	
n-3	0.30^b^	0.30^b^	2.04^a^	0.19	0.000^***^	
SFAs	27.40^c^	57.88^a^	31.36^b^	4.89	0.000^***^	
MUFAs	64.01^a^	29.95^c^	34.99^b^	5.16	0.000^***^	
PUFAs	07.08^c^	19.89^b^	31.43^a,b^	3.58	0.000^***^	

n=4 DF: date fruit, DB: Degla-Beida, WPD: whole pitted date, C: concentrate, SEM: standard error mean, GAE: equivalent gallic acid, DM: dry matter, NS: no significant effect, SFAs: saturated fatty acids, MUFAs: monounsaturated fatty acids, PUFAs: polyunsaturated fatty acids. ^*^ p<0.05 . ^**^ p<0.01 . ^***^ p<0.001 . ^a,b,c,d^ Superscripts indicate statistically significant differences ( p<0.05 ). Concentrate was composed of 60 % corn, 40 % soybean meal, 16 % brain, and 4 % minerals.

The pasture used in the breeding sheep in El Bayadh consists of a variety of small- to mid-sized herbaceous plants well adapted to dry environments, along with an abundant assortment of medicinal flora. Predominant species include wormwood (*Artemisia herba-alba*), esparto grass (*Stipa tenacissima*), albardine (*Lygeum spartum*), bird's-foot trefoil (*Lotus pendaculatus*), white clover (*Trifolium refens*), and saltbush (*Atriplex halimus*), along with species from the families Apiaceae, Fabaceae, Labiaceae, Rosaceae, and Anacardiaceae, and several others such as *Atractylis humilis*, *Calendula arvensis*, *Hordeum vulgare*, *Zizyphus lotus*, *Pistacia atlantica*, and *Capsella bursa pastoris* (Khenfer et al., 2020). The altitude of the grazing areas ranges from 400 to 1200 m, within a semi-arid to arid climate. Annual rainfall varies between 100 to 450 mm, and the region experiences significant thermal fluctuations (Senoussi et al., 2020).

### 2.3 Growth performance

Sheep were separated into two groups and fed one of two diets: a standard concentrate or a diet based on date fruit. The live weight of the lambs was then monitored monthly to assess bodily changes resulting from the dietary substitution. Weights were recorded in the morning for each animal using a needle scale with a maximum capacity of 500 kg and an accuracy of 100 g. Total weight gain (TWG) was ascertained by computing the difference between final body weight (FBW) and initial body weight (IBW).

### 2.4 Slaughter and sampling

Following 12 months of rearing, the lambs were transported for 15 min at slow speed from the farm to a local commercial slaughterhouse (20 km). This short, low-stress journey may have beneficial effects on meat quality. Upon arrival, the animals were allowed to rest for 1 h before being slaughtered. The carcasses were then refrigerated at 4° for 2 h. Subsequently, samples of *Longissimus thoracis* muscle were carefully removed from each carcass, deposited in isothermal containers, and transported to the laboratory for further examination. In the laboratory, these muscle specimens underwent a series of preparation steps including trimming, mincing using a meat grinder, vacuum packaging, and storage at – 20° pending comprehensive analytical evaluation.

### 2.5 Measurements

#### 2.5.1 Diet and meat analysis

To ascertain dry matter and crude ash levels, samples were subjected to heating in a drying oven at 103° for 24 h and 550° for 4 h, respectively (AOAC, 1990). The extraction of total lipid content was performed following the protocol established by Folch et al. (1957). Crude fiber content in the diet was assessed using the method proposed by Van Soest et al. (1991), while crude protein quantification was conducted utilizing the Kjeldahl technique (AOAC, 1990). Furthermore, the total phenolic content in the diets was evaluated based on the method developed by Miliauskas's et al. (2004). The free sugar composition of date kernels was determined by chromatographic methods, specifically GLC and MS according to protocol of Honda et al. (1989). Antioxidant capacity was evaluated using the 2,2-diphenyl-1-picrylhydrazyl radical (DPPH) scavenging assay to measure free radical scavenging activity. This assay was performed according to the protocol described by Brand-Williams et al. (1995). In this procedure, 0.1 mL of sample extract was combined with 3.9 mL of DPPH solution (0.025 g L^−1^) in methanol. The solution was then allowed to stand for 60 min in a dark environment at ambient temperature. After incubation, absorbance at 515 nm was measured using a spectrophotometer. Initial concentration of DPPH in mixture (CDPPH) was determined from a calibration curve, employing the following equation:

1 $$\mathrm{Abs}(515\;\mathrm{nm})=12.509\times (\mathrm{CDPPH})-2.58\times 10^{-3}.$$

#### 2.5.2 Fatty acid profile

In a single reaction vessel, fatty acids were isolated and esterified using an adapted direct methylation technique (O'Fallon et al., 2007). Initially, samples of meat total fat or feed extract were placed in a 15 mL glass tube to which 0.5 mg of tridecanoic acid, 5.3 mL of methanol, and 700 µ L of 10 N potassium hydroxide (KOH) were added. The mixture was then incubated for 1.5 h at 55° in a water bath, with vortexing every 20 min. Once cooled to ambient temperature, 580 µ L of 24 N sulfuric acid (H_2_SO_4_) was added. The sample underwent subsequent cooling and incubation phases. Subsequently, 3 mL of hexane was added to the mixture, which was then subjected to centrifugation at 3000 rpm for 5 min and vigorously mixed using a vortex mixer for another 5 min. The upper layer was carefully transferred to a chromatography vial (Agilent, Santa Clara, CA, USA) and subjected to analysis via gas chromatography. Analysis was conducted on an SP-2560 capillary column (100 m × 0.25 mm internal diameter; 0.2 µ m film thickness, Sigma-Aldrich, St. Louis, MO, USA) using a gas chromatography system with a flame ionization detector (GC-FID, Agilent 7890B, Santa Clara, CA, USA). Peak identification was referenced to FAME 37 Standard Mix. GC-FID conditions were set according to the FAME 37 protocol, with a temperature ramp up to 140° over 5 min, held at 240° for 28 min, and then cooled at a rate of 4° min^−1^. The split ratio was 1:30 , with both the injector and detector temperatures held constant at 260°. An injection volume of 1 µ L was employed. The results were presented as a percentage of total fatty acids detected.

#### 2.5.3 Lipid oxidation

Lipid oxidation in meat samples was evaluated 5 d post-slaughter using a sophisticated third-order derivative spectrophotometric method tailored for detecting malondialdehyde (MDA), an indicator of secondary lipid peroxidation (Botsoglou et al., 1994). Briefly, 8 mL of a 5 g/100 mL solution of trichloroacetic acid (Sigma Aldrich Co., St. Louis, MO) and 5 mL of a 0.8 g/100 mL solution of butylated hydroxytoluene (Sigma Aldrich) in hexane were added to meat samples to achieve homogenization. This was followed by centrifugation, after which the upper layer was discarded. From the lower layer, a 2.5 mL aliquot was mixed with 1.5 mL of a 0.8 g/100 mL aqueous solution of 2-thiobarbituric acid (TBA) (Sigma Aldrich Co., St. Louis, MO) and incubated for 30 min at 70°. Following incubation, the mixture was cooled using tap water before undergoing and being submitted to spectrophotometry in the range of 400–650 nm using a Shimadzu Model UV-160A spectrophotometer (Tokyo, Japan). Third-order derivative spectra were produced by digitally differentiating the standard spectra with a derivative wavelength difference of 21 nm. The concentration of MDA was quantified based on the peak height at 521.5 nm and the linear regression data from the calibration curve constructed using 1,1,3,3-tetraetoxypropane (Sigma Aldrich Co., St. Louis, MO).

**Table 2:** Effect of date fruit supplementation on the growth performance of Hamra lambs.

	Group 1	Group 2	SEM	p value
Body weight at start first month (IBW) (kg)	15.27	17.35	1.22	^*^
Weight at slaughter end 12th month (FBW) (kg)	44.42	52.18	2.64	^**^
Weight gain (WG) (kg/8 months)	29.15	34.83	1.12	^**^

n=50 , Group 1: animals fed on date fruit, Group 2: animals fed on concentrate, SEM: standard error mean. ^*^ p<0.01 significant differences. ^**^ p<0.01 high significant differences.

#### 2.5.4 pH

Using 20 mL of distilled water, 10 g of lamb meat sample was completely homogenized, and a pH meter (Mettler Toledo, SevenEasy, USA) was used to assess the sample's pH (Carse and Locker, 1974). The ultimate pH was measured 24 h post-slaughter. The final pH value was calculated as the mean of three measurements per animal within each group.

#### 2.5.5 Color

Fresh meat color assessment was conducted on a 1 cm thick slice of meat, freshly cut to expose the muscle to air. Following a 30 min period for oxygenation, color metrics were assessed using a Chroma Meter CR-300 and DP-301 data processor (Minolta Co., Ltd., Japan). This evaluation quantified lightness ( $L^{*}$ ), redness ( $a^{*}$ ), and yellowness index ( $b^{*}$ ). Each assay was carried out at least three times per muscle in each group, and all data were reported as mean values.

#### 2.5.6 Vitamin E

Quantification of α -tocopherol in meat and dietary samples was conducted after thawing, with each specimen analyzed in duplicate. The isolation process followed the technique described by Li et al. (1999), utilizing high-performance liquid chromatography (HPLC) for measurement. The HPLC apparatus featured a manual injector, System Gold^®^ interface, and a 114-M pump by Beckman Coulter (Spain), complemented by a fluorescence detector from Jasco (Spain). The setup also included a Kromasil Silica 150 34.6 (5 mm) analytical column and a protective column (10 mm) sourced from KR100-10-10C5 (Symta, Türkiye). The mobile phase, a mixture of isooctane and tetrahydrofuran at a 90:10 ratio, was propelled at a flow rate of 1 mL min^−1^. The detection of α -tocopherol took place at an emission wavelength $\lambda_{\mathrm{em}}$ of 297 nm and an excitation wavelength $\lambda_{\mathrm{ex}}$ of 321 nm. The α -tocopherol content was reported in mg kg^−1^ of lamb, using a dl- α -tocopherol standard from Sigma-Aldrich Chemical (Madrid, Spain) for calibration.

### 2.6 Statistical analysis

The statistical analysis was performed with Minitab software 19.0. A Tukey test was conducted after a one-way analysis of variance. Each experiment was carried out a minimum of three times, and all data are shown as mean ± SD. p values lower than 0.05 denoted statistically significant differences.

## 3 Results

### 3.1 Diet chemical composition

The chemical composition of sheep feed ingredients is presented in Table 1. The results showed more moisture and crude ash contents in the date fruit ( p<0.01 ). However, concentrate feed presented a great amount of crude protein ( p<0.01 ) and total fat content ( p<0.001 ) compared to the other feeding ingredient. Date fruit presents significantly ( p<0.001 ) great amounts of crude fiber and polyphenols compared to the concentrate-based diet (Table 1). The results also indicate that date fruit contained higher levels of vitamin E than the concentrate feed. However, pasture had higher vitamin E content compared to date fruit ( p<0.001 ). Furthermore, the data revealed that polyunsaturated fatty acid (PUFA) levels such as α -linolenic acid C18:3n-3 (ALA) and linoleic acid C18:2n-6 (LA) levels were significantly higher and more predominant in concentrate feed compared to poor-quality date fruit. Oleic acid emerged as the predominant fatty acid in DB dates, recognized as the most extensively distributed natural fatty acid (Table 1).

**Figure 2:**
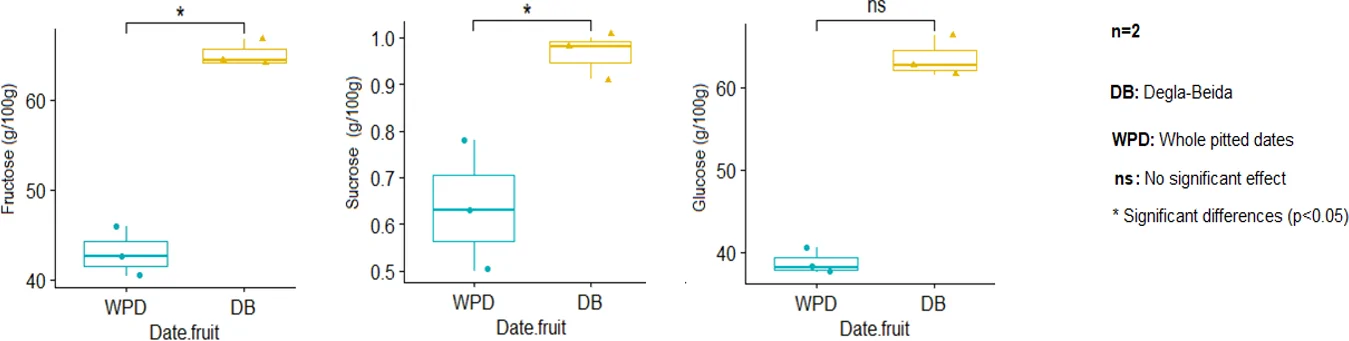
Sugar content in date fruit varieties used for sheep breeding at El Bayadh farm.

Livestock farming in the Saharan region studied uses two varieties of dates, as detailed in Fig. 1. Analysis of sugars highlighted significant differences between the two varieties studied ( p<0.05 ) and none for glucose (Fig. 2). The results in fructose and sucrose contents are more important in whole pitted dates (WPDs), but values are lower in glucose.

### 3.2 Growth performance

The impact of the replacement of concentrate by date fruit on lamb growth performance is documented in Table 2. A significant difference was observed in the initial weights of lambs across the different rearing systems. The integration of concentrated feed significantly ( p<0.01 ) influenced the final weight at slaughter and exhibited superior weight gain ( p<0.01 ) compared to the group fed date fruit.

**Figure 3:**
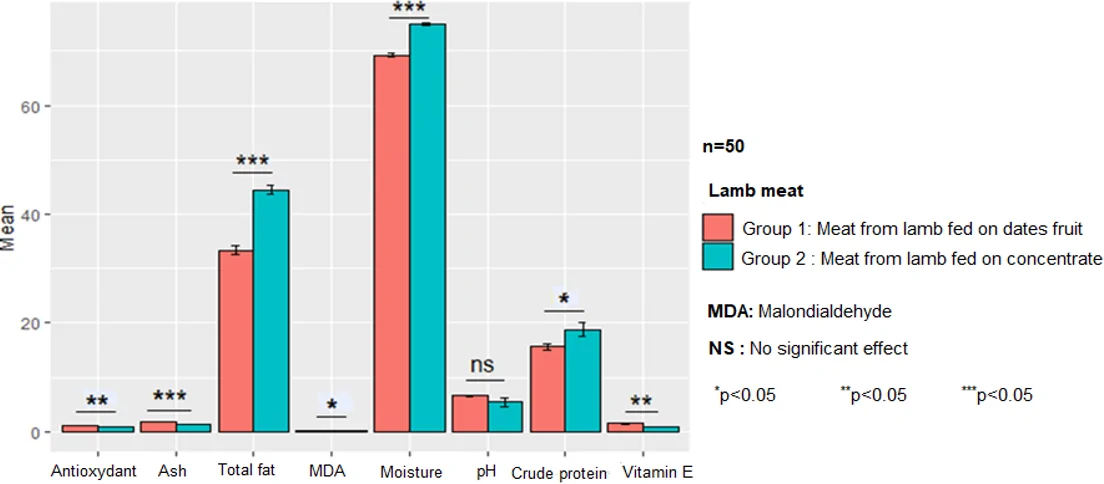
Effect of feeding diet on *Longissimus thoracis* muscle composition.

### 3.3 Meat composition

As shown in Fig. 3, replacing concentrate feed with date fruit had a significant impact on meat composition, but none on meat pH. Results showed more fat content and crude protein ( p<0.001 ) in meat from lambs grazed in Group 2 and receiving concentrated feed in their rations compared to lamb meat from the other one. However, the analysis of crude ash in meat from both groups shows a predominance for the date fruit group rearing system ( p<0.001 ). Additionally, meat from lambs receiving date fruit feed in their ration was richer in vitamin E compared to lamb meat supplemented with only concentrate ( p<0.01 ). Moreover, it was observed that WPD and DB decreased ( p<0.05 ) the lipid oxidation of studied muscles significantly (Fig. 3).

### 3.4 Meat fatty acids

**Figure 4:**
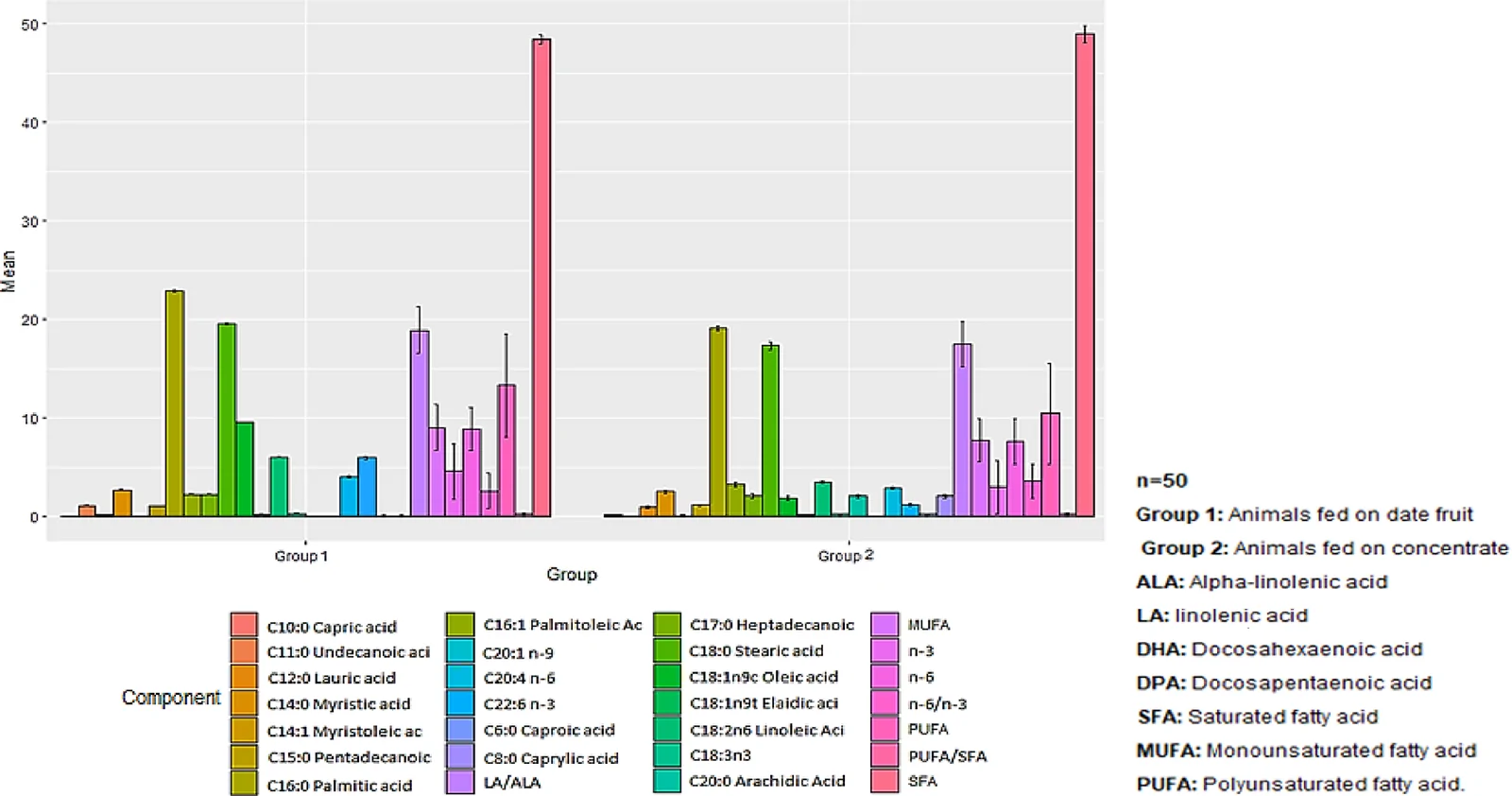
Fatty acid profile of meat of lambs (% of the total fatty acid identified).

Figure 4 outlines the fatty acid composition in lamb meat. Differences in saturated fatty acid (SFA) levels were observed between lambs fed with date fruit and those without supplements. Notably, palmitic acid (C16:0) and stearic acid (C18:0), the primary SFAs, showed increased concentrations in lambs from Group 1 when compared to those from the other one, with these differences being statistically significant. Additionally, PUFAs exhibited a marked increase. Specifically, the concentrations of linoleic acid (C18:2n-6), linolenic acid (C18:3n-3), arachidonic acid (C20:4n-6), and docosahexaenoic acid (C22:6n-3) were significantly higher ( p<0.001 ) in lambs' meat fed on date fruit (Fig. 4). Furthermore, the n-6 / n-3 ratio in the meat of lambs on a concentrate diet was considerably higher compared to that in the group supplemented with date fruit ( p<0.001 ).

### 3.5 Meat color

Significant variations ( p<0.001 ) in meat color were recorded (Table 3), with notable predominance for the luminosity and particularly for red index in the group of lambs fed on date fruit, indicating a marked difference.

**Table 3:** Effect of date fruit supplementation on meat color of the Hamra lamb breed.

	Group 1	Group 2	SEM	p value
$L^{*}$	59.60	48.68	1.89	0.000^***^
$a^{*}$	09.36	05.13	0.04	0.000^***^
$b^{*}$	14.88	09.24	0.09	0.000^***^

n=50 , Group 1: animals fed on date fruit, Group 2: animals fed on concentrate, SEM: standard error mean, $L^{*}$ : luminosity, $a^{*}$ : redness, $b^{*}$ : yellowness. ^***^ More highly significant differences ( p<0.001 ).

## 4 Discussion

The region of El Bayadh, where the study was carried out, is characterized by limited natural resources, poor soil, sparse plant formations, and harsh climatic conditions. Consequently, date fruit represents a suitable feed supplement in place of concentrate. Their use was aimed to balance the animals' ration more effectively. As reported in Table 1, the basic ingredients of the diet (date fruit in Group 1 vs. concentrate feed in Group 2) differ in their chemical composition based on the analysis of each specimen. WPDs contain higher levels of moisture and crude fiber but lower levels of crude ash, total fat, and protein contents. According to research by Ghnimi et al. (2017), the chemical profile of date fruit is contingent on the crop variety. Notably, the crude protein levels in DB dates were below the 5 %–7 % range noted by Galab et al. (2021), which was not the case for the WPD variety. This variance could be attributed to differences in tissue weights and densities among varieties or possible disparities in nitrogen fertilizer application. Date fruit is significantly ( p<0.001 ) richer in polyphenols compared to the concentrate-based diet. The DB dates' variety exhibited the highest concentration of total polyphenols that, in both types of date fruit, fall within the range identified by Hassan (2021), who documented phenolic content in 10 Algerian date varieties, with values ranging from 226 to 955 mg of gallic acid equivalent per 100 g. Moreover, lower levels of crude fiber were observed in concentrate feed and DB compared to those in WPDs. The concentrate contains higher crude protein and fat contents compared to date fruit. According to Akasha et al. (2012), dates are low in protein but rich in other essential nutrients, including fiber, vitamins, and minerals. Few studies have been conducted on the FA characteristics of date fruit. Among the most abundant saturated acids in date fruit are lauric acid (C12:0), myristic acid (C14:0), palmitic acid (C16:0), and stearic acid (C18:0) compared to concentrate feed (Table 1). In contrast, concentrate contains higher levels of linoleic and linolenic acids. WPDs contained a lower proportion of PUFA than the concentrate feed, likely influenced by genetic, seasonal, edaphic, and climatic variations (Riley et al., 2022).

The carbohydrate composition of date fruit includes soluble sugars like glucose, fructose, and sucrose, along with dietary fibers such as cellulose, hemicelluloses, pectin, and fructans, which are vital components (Al-Harrasi et al., 2014). As depicted in Fig. 2, the WPD variety showcased the highest sugar content compared to DB dates. However, sugar levels can fluctuate among cultivars even at the same stage of maturity, affected by environmental factors that alter nutritional properties. This rich carbohydrate content renders date fruit a valuable source of nutrition and energy. Prior studies have highlighted that fructose and glucose are the predominant sugars in dates, with minor variations in sucrose levels across different varieties (AlShwyeh and Almahasheer, 2023). For ruminants, sugars are a crucial energy source, essential for rumen microflora. Dietary sugars, along with readily available proteins and non-protein nitrogen, facilitate the synthesis of microbial protein in rumen, a process critical for enhancing the nutritional value derived by the animal. This microbial protein is then digested by sheep in the abomasum and hindgut, underscoring the importance of maximizing microbial protein production to improve animal feed efficiency (AlShwyeh and Almahasheer, 2023).

Over a period of 10 months, lambs from Group 2 exhibited greater weight gain (Table 2), a phenomenon that can be attributed to the high-energy and protein-rich content of the feed concentrate. These results align with those reported by Sabry et al. (2021), who observed that varying concentrate levels in lamb diets can improve profitability. The notable weight increase is likely due to improved feed digestibility, which might stem from legumes' presence such as *Lotus pendaculatus* and *Trifolium refens* in El Bayadh pasture. These legumes are known to enhance rumen fermentation and boost nutrient absorption. Additionally, natural grazing practices foster a healthy balance between rumen bacteria and protozoa, the latter being recognized for their role in enhancing protein degradation (Ushida and Jouany, 1985). Additionally, incorporating feed concentrates such as corn and soybean into Group 2 farming strategies enhances lamb weight gain. Ríos-Rincón et al. (2014) highlighted that protein-rich diets correlate positively with better growth performance, due to the rejuvenation of muscle proteins. This aspect is economically pivotal in meat production. Moreover, achieving an optimal balance of muscle protein retention throughout the animal's life with minimal feed intake (indicating a higher feed conversion rate) is critical for reducing environmental impact during production. The substitution of concentrate with date fruit influences body weight gain. According to Ríos-Rincón et al. (2014), diets enriched with up to 30 % date residue effectively support growth and nutrient assimilation in lambs without detrimental effects. Hassan (2021) further affirms that integrating up to 50 % date fruit in feed rations positively affects daily weight gains and ultimate body weight in sheep.

The primary determinant of meat acceptance among consumers lies in its quality, which is assessed through various physicochemical characteristics such as intramuscular fat content. The pH level serves as a critical metric for assessing meat quality during carcass maturation. pH measurements in meat from lambs that fed on dates did not significantly exceed those in the other group (Fig. 3). This result is in agreement with Li et al. (2024), suggesting that date waste might accelerate glycolysis, thereby speeding up pH reduction in meat. Despite variations in feeding systems, their impact on meat pH was minimal. Meat from lamb fed on date fruit displayed higher crude ash content compared to lamb meat fed on concentrate. This can be attributed to the quantity and type of supplements used in the present study. Date fruit rich in carbohydrates and minerals such as calcium, potassium, and phosphorus, presents a viable energy source for ruminants. Sabry et al. (2021) demonstrated that elevated concentrate levels significantly augmented the dimensions of ruminal papillae compared to those in groups fed with hay and lower concentrate levels. The rumen serves as the primary site for bacteria to break down plant materials into volatile fatty acids and microbial proteins, a process heavily influenced by the dietary energy source and its concentration (Xu et al., 2021). The total lipid content in meat of lambs fed date fruit in Group 1 is significantly lower than that of animals fed concentrate in Group 2. This difference may be attributed to the low-fat content of the date fruit under study, as seen in Table 1. The quantity and type of lipids accumulated in muscle are primarily determined by dietary conditions, digestion processes, intestinal absorption, liver metabolism, and the lipid transportation system to the muscle. Consumer acceptance of meat primarily hinges on its quality, discerned through physicochemical properties such as intramuscular fat content. This trait is affected by variables such as species and breed of animal, dietary practices, body weight at the time of slaughter, type of muscle, and aging period of meat (Karaca et al., 2016). Vitamin E is produced exclusively by plants, specific algae, and cyanobacteria (Mène-Saffrané, 2018). It is essential for animals and must be obtained through their diet. This vitamin serves as a vital antioxidant, protecting polyunsaturated fatty acids and proteins from oxidative damage. Despite a broad agreement that high doses of vitamin E do not enhance the growth or carcass quality of light lambs (Li et al., 2024), its significant role in mitigating color degradation, preventing off-odors, and protecting against lipid oxidation is well recognized. Meat from lambs fed date fruit displayed elevated levels of vitamin E compared to the meat of the animals fed a concentrate diet. Increasing α -tocopherol content in meat can be accomplished by supplementing ruminant diets with vitamin E at quantities that surpass their nutritional requirements, as suggested by NRC (1985) (15 to 40 mg kg^−1^ of feed). It is essential to maintain an optimal α -tocopherol level to improve preservation of lamb meat. Research by Álvarez-Rodríguez et al. (2022) suggests that an α -tocopherol range of 1.07 to 2.37 mg kg^−1^ meat effectively reduces lipid and pigment oxidation over extended storage periods. The influence of vitamin E on meat quality hinges on its concentration in muscle tissue, which varies based on form and amount of vitamin E consumed, the length of feeding program, and specific type of lamb (light or heavy). According to antioxidant activity assessments in this study, meat from lambs fed on date fruit exhibited superior antioxidant activity compared to that from concentrate-fed lambs. This improvement is attributed to elevated levels of phenolic compounds in whole pitted dates (WPDs) and Degla-Beida (DB) dates compared to concentrates. Phenolic compounds, a major class of natural antioxidants, positively impact lamb meat by improving the fatty acid composition and reducing MDA levels. Oxidative processes in muscle foods result in the breakdown of lipids and proteins, reducing nutritional value and presenting significant hurdles in the development of new, convenient meat products, ultimately influencing consumer acceptance. Analysis indicated that lamb meat from animals fed with concentrates has a comparatively high fat content, which primarily contributes to quality decline through lipid oxidation ( p<0.05 ), as demonstrated by associated thiobarbituric acid reactive substance (TBARS) values (Fig. 3). These results highlight a strong antioxidative action in minimizing hydroperoxide formation during storage, consistent with findings by Abdel-Hamied et al. (2009), who observed that diets rich in phenolic compounds, such as sage and their combinations, possess robust antioxidative capabilities during both refrigerated and frozen storage of minced meat.

The disparities in the fatty acid profiles between lamb meat from both groups are attributed to the differences in their feed compositions, specifically between the concentrate feed and by-products utilized. Several production variables such as breed, age, and dietary formulation are key determinants of fatty acid profiles in lamb meat (Xu et al., 2021). Notably, substituting traditional concentrates with date fruit increased concentrations of MUFA and PUFA in meat (Fig. 4). This rise is largely attributed to the substantial presence of unsaturated fatty acids, particularly oleic acid (C18:1n-9c), in WPD used as a sustainable feed, confirming studies by Mousa and Zedan (2008). Key fatty acids identified in the meat from lamb fed on date fruit include palmitic acid (C16:0), stearic acid (C18:0), and oleic acid (C18:1n-9c). Conversely, lambs that consumed a protein-rich diet exhibited elevated levels of SFA, which can be explained by the fact that the Algerian dietary habit is based on the consumption of concentrate-based protein sources like corn, soybean meal, brain, and minerals, as well as meat, which are considered protein sources and also sources of saturated fats in comparison to lambs fed on date fruit, with the dietary approach having little influence on meat SFA levels. In both groups, a decline in MUFA, particularly C18:1, was observed alongside an increase in PUFA. This indicates a shift towards greater intramuscular fat deposition relative to the SFA ratio, potentially influenced by varying dietary fiber contents. The concentrations of linoleic acid (C18:2n-6), linolenic acid (C18:3n-3), arachidonic acid (C20:4n-6), and docosahexaenoic acid (C22:6n-3) were significantly higher in the meat of lambs fed date fruit compared to the meat of animals fed only a concentrate diet. The concentrate proportion in the diet also resulted in decreased levels of n-6, n-3, and LA / ALA ratio in the lamb's *L. thoracis* from Group 2. Inclusion of antioxidant-rich by-products in diet leads to increased daily consumption of ALA and total n-3 PUFA in meat (Karaca et al., 2016). In terms of the PUFA/SFA ratio, meat from lambs consuming DF displayed a markedly higher ratio than that of lambs consuming a higher level of concentrate, as identified by O'Fallon et al. (2007). They noted that feeds based on concentrate, which are higher in C18:3n-3, subsequently boost PUFA levels, DPA, EPA, and DHA in meat.

Meat color significantly impacts consumer perceptions of quality, influencing their assessment of other crucial properties such as flavor and aroma. Higher variations in meat coloration were recorded, emphasizing the integral role of diet in defining meat's visual appeal. These findings are consistent with those reported by Ruedt et al. (2023), who proposed that an increased concentration of polyphenols adversely affected coloration of lamb meat reared on DF, as indicated by elevated color metrics. Additionally, meat from lambs raised on low-quality date fruit exhibited a higher degree of brightness compared to that from animals fed only on concentrate feed. This is likely caused by protein denaturation, which increases dispersion of light. According to Bekhit et al. (2019), different animal parameters (breed, age, sex, food, activity level, and species), as well as the kind of muscle, determine the variation in flesh color. Regarding the chromatic coordinate $a^{*}$ , which designates redness, the analysis revealed that meat from the group fed date kernels consistently exhibited higher $a^{*}$ values compared to the meat from lambs fed a concentrate diet. These variations are predominantly influenced by the oxidation of myoglobin. Additionally, the presence of certain PUFAs can further impact meat color (Ruedt et al., 2023).

## 5 Conclusions

Date fruit, as a high-energy supplement and an economical alternative to conventional concentrate, can be valorized as a beneficial ingredient for small ruminants, improving the nutritional characteristics of meat and reducing their lipid content with these added natural compounds. This meets consumer preferences and purchase intention. Indeed, their use proved to be a sustainable and cost-effective solution that can have a favorable impact on the economy of regions such as the Saharan pastoral areas of Algeria. Despite the slightly lower growth rates, the lambs fed date fruit produced meat with a healthier fatty acid profile but also a boosted vitamin E content, which helps reduce lipid oxidative stability. The meat from these lambs showed a notable increase in PUFA, including omega-3, which enhances the nutritional quality of the meat and makes it more appealing to health-conscious consumers. Overall, the study highlights that incorporating agricultural by-products such as date fruit into livestock diets presents a valuable strategy for promoting sustainability and improving meat quality. The results suggest that, particularly in arid regions with limited natural resources, supplementing traditional feed with date fruit can help reduce feeding costs, enhance the nutritional profile of meat, and support circular agricultural practices. However, in order to make the present study practicable in the real economy, further studies need to be conducted to set up a suitable protocol to balance growth performance with the beneficial characteristics of lamb meat and to assess organoleptic quality including the aroma profile of the lamb meat.

## Data Availability

The datasets used in this study are available upon request from the authors.
